# Fractionation of wood due to industrial chipping: effects and potential for Kraft pulping of European spruce

**DOI:** 10.1007/s10570-024-05804-0

**Published:** 2024-03-03

**Authors:** Roman Poschner, Caterina Czibula, Adelheid Bakhshi, Thomas Harter, Rene Eckhart, Ulrich Hirn

**Affiliations:** Institute of Bioproducts and Paper Technology, Graz University of Technology, Inffeldgasse 23, 8010 Graz, Austria

**Keywords:** Chip size, Yield, Screening, Fiber length, Spruce

## Abstract

The research conducted on kraft cooking of for different chip sizes is often not representative for the industrial process since the chip size fractions were made of high-quality wood without impurities. We evaluated the effects and the potential of cooking non ideal spruce chip fractions after industrial chipping and screening. The chips were classified according to SCAN 40:01, and the respective fractions were cooked under the identical conditions to mimic the effect of a joint cooking in the industrial digester. For the undersized chips we found higher bark content, a lower screened yield, a higher Kappa number, lower fiber length and lower tensile strength. For the oversized chips, the fiber length and tensile index were also considerably lower. A lower wood quality due to high knot content in the larger fractions was found to be the reason for that. Based on the data obtained from the experiments and literature, different process options for increased yield and reduced chemical consumption are discussed, e.g., separate cooking of different chip fractions. Improved chip screening seems to be the process improvement with lowest costs and highest impact.

## Introduction

Due to the large scale of the industrial Kraft pulping process improvements in terms of yield or resource efficiency are having a large economical and ecological impact. In addition to cooking parameters such as temperature, time, effective alkali or liquor-to-wood ratio, chip dimensions are considered to be of vital importance in kraft pulping. Several studies focused on the influence of chip size at laboratory scale, with chip thickness emerging as a critical value (Hartler and Onisko 1962; Gullichsen et al. 1992; Tikka et al. 1993). Based on data from laboratory experiments, modeling approaches were undertaken to understand the kinetics of the process and its dependence on chip geometry (Akhtaruzzaman et al. 1980; Gustafson et al. 1983; Dang and Nguyen 2008).

Experiments were usually conducted with laboratory made wood chips, in which logs were cut to the desired chip dimensions and then cooked under specific conditions. A chip thickness of about 3 mm or 1/8 inch proved to be favorable for kraft pulping as it provides the best results in terms of yield, screenings, pulp homogeneity and pulp properties (Borlew and Miller 1969). In kraft pulping, a distinction is made between total yield, i.e., the total amount of pulp produced, and screened yield, i.e., the amount of pulp that passes through the screening process. During the screening, rejects like knots and shives are removed from the pulp. Knots are incompletely cooked wood chips, often caused by insufficient impregnation of large/oversized wood chips or dense wood pieces. Shives are incompletely disintegrated or cooked fiber bundles. According to Quinde, the ideal chip dimensions are 25 mm in length and 4 mm in thickness (Augusto Quinde 2020a, b). Thicker or larger chips tend to result in higher total yield, but at the same time in an increased rejects content and lower screened yield (Colombo et al. 1964). Thinner or smaller chips are overcooked in the process when treated under the same conditions. This results in lower Kappa and reject content, but also in lower total- and screened yield (Gullichsen et al. 1992).

On an industrial scale, logs are not cut to a specific chip size, but to a target chip thickness. Smaller and larger chips are produced inevitably. According to the work of Akhtaruzzaman et al., the difference between industrial and handmade chips is less pronounced in smaller chip fractions (Akhtaruzzaman and Virkola 1979b). However, as chips get thicker, deviations become observable. Larger chips are subjected to high mechanical stress during industrial processing. These stresses cause the chips to break, partially delaminate and leave cracks on their surface (Hatton and Keays 1973). Consequently, the impregnation behavior of thick industrial chips is better than that of handmade chips of the same size. To produce the same amount of rejects from 4.5 mm laboratory chips, technical chips with a thickness of 10 mm are required (Hatton 1975).

In more recent literature (Luu and Shariff 2003; Morton et al. 2012), technical chips from pulp mills were classified according to their thickness, prior to laboratory kraft cooking. Thick fractions (> 8 mm) turned out to be undercooked, which resulted in a high rejects content and low screened yield. Thin fractions (< 2 mm) were overcooked, which resulted in no rejects but a slightly lower screened yield. Similar results for the reject rates were found in other publications (Becker 1992). Although fines end up in the digester on an industrial scale, they were not included in those experiments.

In the process of chipping, significant amounts of undersized and oversized chips are produced, passing through screens and entering the digester (MacLeod 2007). MacLeod noted that it may be advantageous to increase yield by using a separate equipment for digesting undersized fractions. Further, mechanical treatment like crushing of the oversized fractions is used for kraft cooking.

In this work we are determining effects and the potential of using oversized and undersized wood chip fractions in separate production lines. In contrast to the existing literature, we are focusing on industrial chips from a pulp mill. A chip sample from a European pulp mill was classified, and the individual size fractions were lab-cooked under the same cooking conditions. The resulting pulp was analyzed for yield, fiber length, Kappa and tensile strength. Based on that data process recommendations for more efficient pulping are given.

## Materials and methods

### Chip sample preparation and chip size analysis

**Fig. 1:**
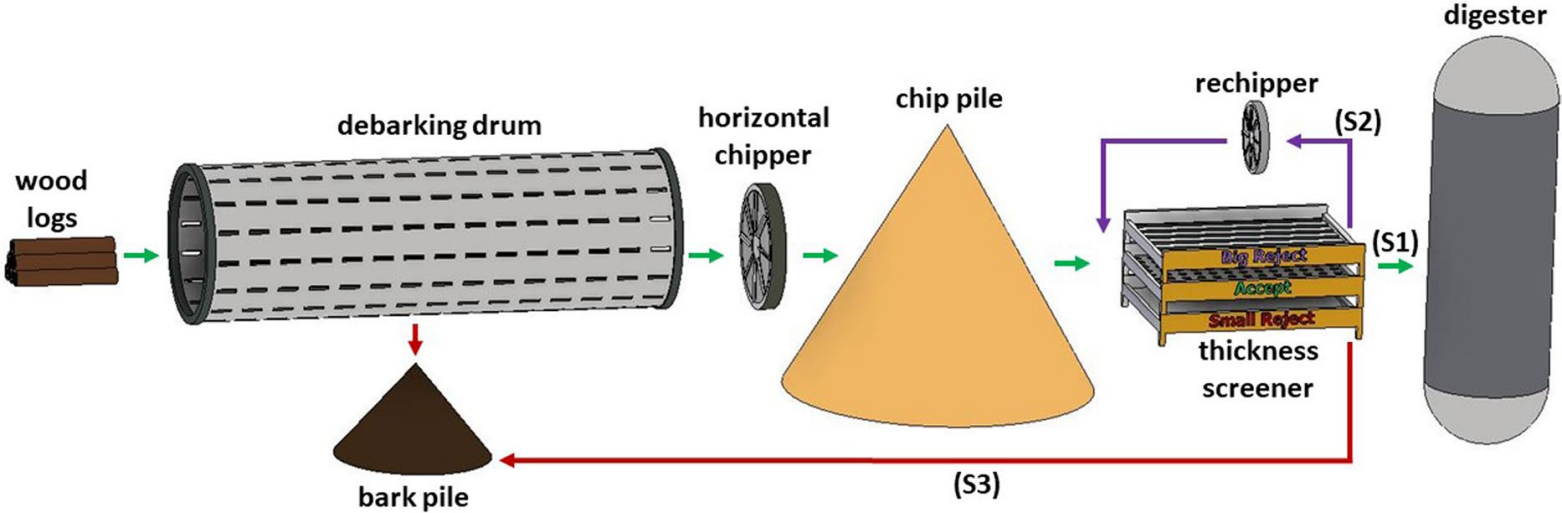
Wood handling process scheme of the pulp mill. After thickness screening the chips are divided into 3 streams: accept stream (S1), coarse reject stream (S2) and fine reject stream (S3). The wood chips for this work were sampled from the S1 accept stream

Spruce chips were industrially produced and provided by a European industrial pulp mill. A simplified process scheme of chip production is illustrated in Fig. 1. Logs of spruce are debarked on a debarking drum which has an average yearly debarking loss of 0.86%. The debarked logs, having an average dry content of 62.14%, are chipped on a horizontal chipper utilizing turn knives. The knives are adjusted to give an average chip thickness of 4 mm. The chips are then transported to the chip pile for storage. Before the spruce chips are processed in the digester, they are classified into coarse (S2–retained by 7.5 mm slots), accept (S1–passing through 7.5 mm slots, retained 6 mm squares) and fine fractions (S3–passing through 6 mm squares) by screening. The fine reject is transported to the bark pile while the oversized rejects are shredded in the re-chipper to reduce their size (to a thickness of approximately 4.5–5 mm) and, subsequently, reentered the screening process.

**Fig. 2:**
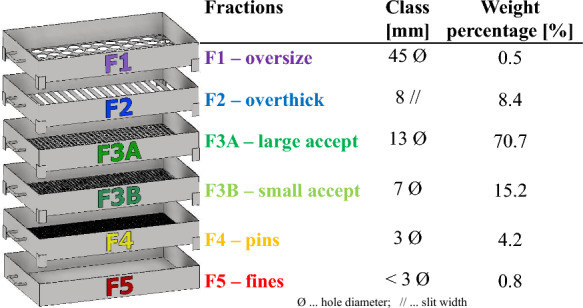
Utilized screens for classification are shown next to the chip fraction nomenclature. Hole sieves with specified diameter Ø and slots with specified thickness//were used. Rightmost column gives the mass fraction of the individual size classes. The color coding of the fractions is used throughout this work

The provided online measurement data of the chip dimensions were recorded by an Andritz ScanChip system both after the chipper as well as after the thickness screening. The chip sample for the experimental part of this work was taken after the screening and included solely the accept fraction (S1) which is going from the pile to the digester in the industrial process. The chips were subsequently screened into their respective size fractions according to SCAN 40:01 on a Lorentzen & Wettre laboratory screener. The size of the screen openings and an illustration of the screening apparatus are presented in Fig. 2. The rightmost column in Fig. 2 gives the mass fraction of the individual size classes as determined by the screening.

**Fig. 3:**
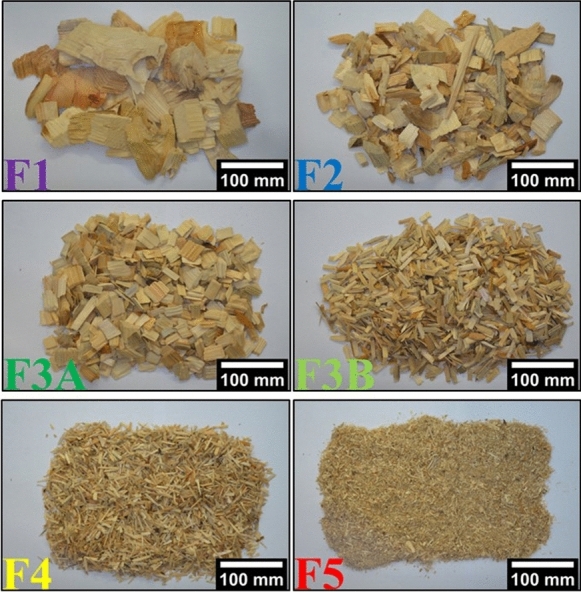
Chip fractions obtained by the classification: F1 oversized/F2 overthick (top), F3A large accept/F3B small accept (middle), F4 pins/F5 fines (bottom)

The chip fractions, presented in Fig. 3, consisted of oversized chips (F1—retained by 45 mm holes), overthick chips (F2—retained by 8 mm slits), large accept chips (F3A—retained by 13 mm holes), small accept chips (F3B—retained by 8 mm holes), pins (F4—retained by 13 mm holes) and fines (F5—passing through 3 mm holes). The chips were stored in paper bags in a room (at ~ 50% relative humidity; T =  ~ 23 °C) for approximately one month to equalize their moisture content, reaching 93% solids content (ISO 638:2008).

### Cooking of chips

**Fig. 4:**
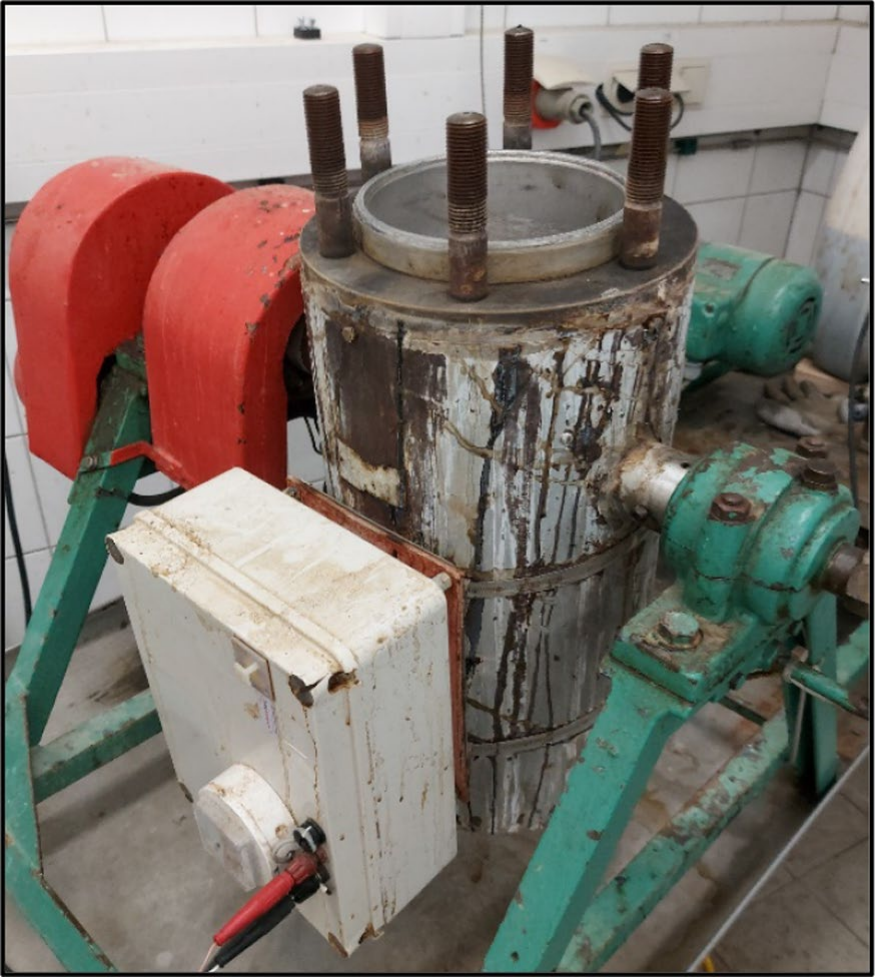
Rotary digester used for the experiments. The reactor lid is not present in the picture

Fraction F3A was used in pre-trials to optimize the cooking parameters in the rotary digester, which is depicted in Fig. 4. Several trial cooks were performed to achieve a kappa number of 60 while also keeping the final pulp properties as close to industrial standards as possible. Steaming of the chips was achieved by evaporating water inside the closed reactor vessel. Once the set temperature of 105 °C was reached, the atmosphere in the system was enriched with steam by venting the vessel for a few seconds. The ramp-up time to reach the set temperature for steaming was 23 min and the hold time at this temperature was 20 min. After the steaming phase, the reactor was opened to fill in the required amount of white liquor and additional water. Then, a ramp-up time of 27 min was required to reach the desired temperature of 110 °C for impregnation, which was held for 20 min. The cooking temperature of 165 °C was reached after 35 min and was held for 90 min. Cooling below 100 °C was done by slowly venting the system for approximately 20 min. Ramp and hold times were automated and adjusted using an electronic controller. The digester was rotating 4 times per minute.

All chip fractions were cooked identically using the cooking conditions found in the optimization pre-trials for Fraction 3A and are shown in Table 1. An H factor of about 1000 was obtained with the given settings. Two cooks per chip fraction were performed to evaluate the stability of the process and to achieve reliable results. After the cooking process, a sample of black liquor was taken from inside the vessel and the chips were defibrated in hot water for 10 min using an L&W disintegrator at 3000 rpm. Then the pulp was washed with hot water. Knots and shives were separated using a modified Brecht-Holl fractionator (based on Tappi standard T275), equipped with 2.5 mm hole and 0.39 mm mesh screens. Yield was determined by weighing the total amount of pulp produced and dividing its dry weight by the dry weight of the wood put into the digester.

**Table 1:** Parameters of the individual process steps: steaming, impregnation and cooking; showing the ramp time, hold time and temperature

	Ramp time [min]	Hold time [min]	Temperature [°C]
Steaming	23	20	105
Impregnation	27	20	110
Cooking	35	90	165
Liquor to wood ratio (mass:mass)		7:1	
Effective Alkali charge (wt% on wood)		19%	
Sulfidity		34%	
Mass of wood per cook		800 g (oven dry)	

The table shows the used liquor to wood ratio, effective alkali charge, sulfidity of the white liquor, the mass of chips used per cook and number of cooks per fraction

### Pulp and paper analysis

The standards followed for laboratory beating, laboratory hand sheet preparation and conditioning are listed in Table 2. The pulp and paper properties like dry matter content, fiber length and tensile properties were analyzed according to the standards discussed below.

**Table 2:** Standards used for pulp and paper analysis

Kappa	ISO 302
Determination of dry matter content	ISO 638:2008
Determination of fibre length	ISO 16065-2:2014
Laboratory beating	ISO 5264-2:2011
Preparation of laboratory sheets	ISO 5269-2:2004
Standard atmosphere for conditioning	ISO 187:1990
Determination of tensile properties	ISO 1924-3:2005

Kappa measurements were performed according to ISO 302 and with an automated Fourier transform near-infrared spectroscopy analyzer (FT-NIR) from FITNIR. Two cooks per chip fraction were performed and analyzed separately.

For wood analysis, the chip fractions were ground to a particle size of 200 µm with a Retsch ZM 200 mill. The powder was analyzed for its ash content according to TAPPI T211. The brightness (at 457 nm) of the wood powder was evaluated by mechanically pressing it into tablets and subsequent brightness analysis with an L&W Elrepho color measurement instrument (based on ISO 2470-2:2008). The cross-sections of undercooked wood particles, which are shown in Fig. 8b, were inspected by embedding the particles in resin and microtome-cutting the embedded sample with a diamond knife. Then, an image of the cross-section was recorded by optical microscopy (Wiltsche et al. 2011; Lorbach et al. 2012).

## Results and discussion

In the following section the results of the investigated chip fractions—oversized (F1), overthick (F2), large accept (F3A), small accept (F3B), pins (F4), and fines (F5)—are presented. The results of the chip size analysis, repeatability of the cooks, yield and Kappa, fiber length and paper strength are discussed. Further, difficulties of pulping the fines fraction, bark impurities in smaller chip fractions and the knot content in larger chip fractions are addressed.

### Chip size analysis after the chipper and screener

**Fig. 5:**
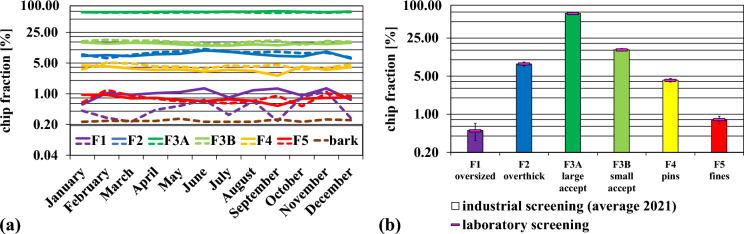
(**a**) Weight percentage of chip fractions recorded by Andritz ScanChip system over the year 2021 (logarithmic scale). The continuous lines represent the chip fractions after the industrial horizontal chipper, dashed lines represent the chip fractions after the industrial thickness screener (S1 in Fig. 1). The amount of bark remaining in the chips after the screener is depicted as a brown dashed line. (**b**) The averaged weight percentage of the chip fractions after the industrial screening over the whole year 2021 is shown as columns, while the solid pink markers represent the results from the laboratory screening of the chips used in the experiments (logarithmic scale). The error bars represent a 95% confidence interval

The results of the chip size analysis for the entire year 2021, recorded with an Andritz ScanChip system at the pulp mill, are presented in Fig. 5a. The values stated here are the monthly averages over the year 2021. The solid lines show that quite large amounts of oversized- and overthick chips (1.0% F1 & 7.9% F2), but also pins and fines (3.7% F4 & 0.8% F5) were produced during chipping. The majority of chips after the chipper consisted of large accept and small accept chips (73.0% F3A & 13.6% F3B). The screens upstream of the digester were not able to separate larger and smaller wood chip fractions effectively, so the majority still entered the cooking process. The best separation efficiency was achieved for the oversized chips (F1), decreasing their relative amount to 0.4%. After the screening, the proportion of the overthick fraction (F2) increased to 8.6%. Surprisingly, the relative amount of the large accept fraction (F3A) decreased to 70.7%. The chip mixture S1 after screening had an increasing content of the small accept fraction and pins (15.3% F3B and 4.3% F4). The breaking of larger chips into smaller pieces due to mechanical impacts during transport and processing, and storage on the pile should be the reason for that. The number of fines in S1 stayed approximately constant (0.8% F5) compared to after the chipper. The brown dashed line in Fig. 5a shows that the chips were slightly but consistently contaminated with bark throughout the entire year with an average of 0.24%. The bark contamination was quite low and the variation over the year was very stable compared to the variations often reported in the literature (Hart 2009), considering that debarking is much more difficult in winter than in spring or summer.

The values for the laboratory screening of the chip sample drawn for this work are compared to the average results for industrial screening at the mill for the entire year 2021. The overall ratio of the chip fractions from the laboratory screening (0.5% F1, 8.4% F2, 70.7% F3A, 15.2% F3B, 4.2% F4, 0.8% F5) agreed very well with the 2021 results from industrial screening, demonstrating that our chip sample is representative for the industrial process in the mill investigated.

The results also show that under the given circumstances, the separation of nonideal chip fractions in the industrial process was not very efficient for the mill investigated.

### Repeatability of the experiments

**Fig. 6:**
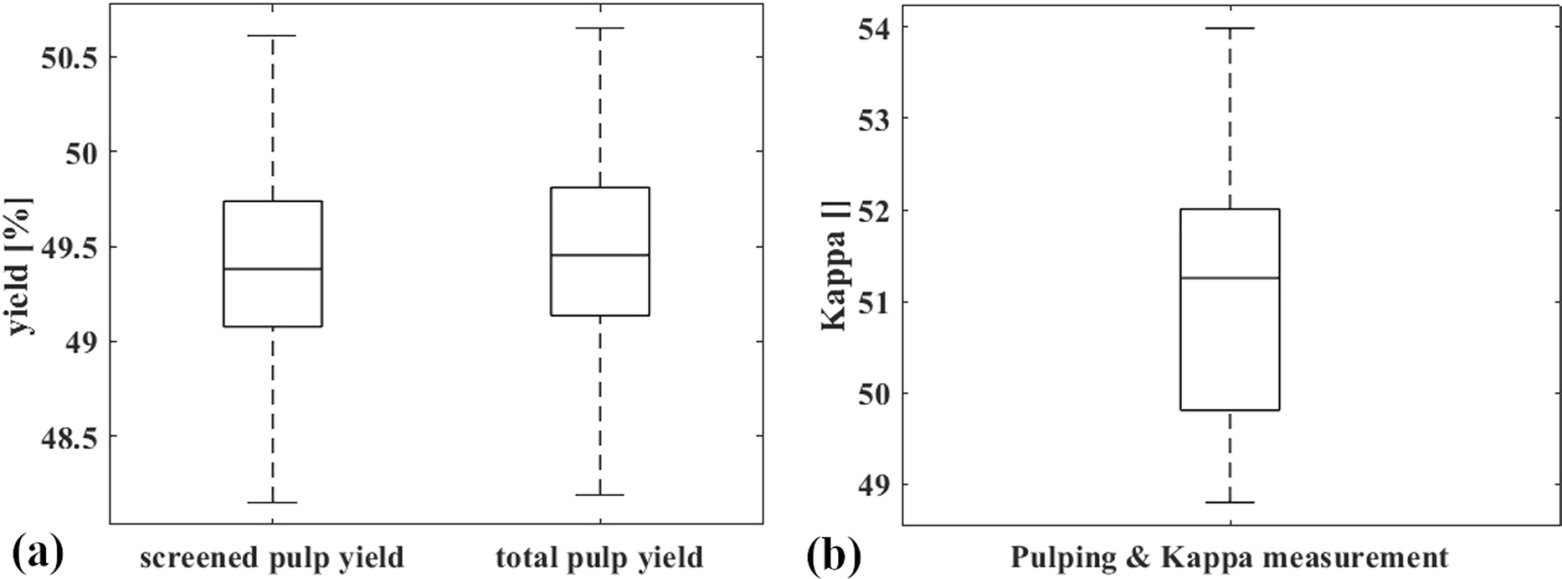
Repeatability boxplots of 6 identical lab cooks. (**a**) Screened pulp yield (left) and total pulp yield (right). (**b**) Kappa

The repeatability of the lab cooking procedure was evaluated by repeating six cooks of the large accept fraction (F3A) under the conditions described in Table 1, but using a white liquor with 38% sulfidity. The results for the yield and kappa analysis of those cooks are shown in Fig. 6. Figure 6a indicates that the total- and screened yield of fraction F3A were very similar, as hardly any knots and shives were produced under these cooking conditions. Figure 6b depicts the variation of Kappa. The data shows that the Kappa value between the cooking experiments has a higher variation than the yield. Please note that these results are caused by the variation of the pulping process *plus* the variation of the Kappa measurement, which is probably one of the causes why the variation of the Kappa value was higher.

Overall, the repeatability of Kappa and yield measurements shows that differences of 3.69 Kappa points (2σ) and 1.87% yield (2σ) between individual cooking trials are variations that are descending from pulping and measurement methods. When comparing results from different cooking procedures, at a confidence level of 95% we have to expect an uncertainty of ± 1.96σ/√n for the resulting values of Kappa and yield (n being the number of repeats of the cooking trial). So, comparing differences between individual cooks (n = 1), need a Kappa difference of and 3.61 and a yield difference of 1.83 to confirm a statistically significant difference. For two repeat cooks, like in this work, the threshold for statistical significance is a difference of 2.56 Kappa points or 1.30% yield. We would like to point out that this variation is not uncommon at all for lab pulping investigations.

The main factors influencing the repeatability of the cooks in our opinion are the sampling of wood chips, the kraft pulping in the rotating digester (mixing of the liquor and chips), the downstream pulp processing (separation of shives and knots form the pulp) and the sampling of the pulp. Finally, the uncertainty of the measurement method is also contributing.

### Yield and Kappa results

**Fig. 7:**
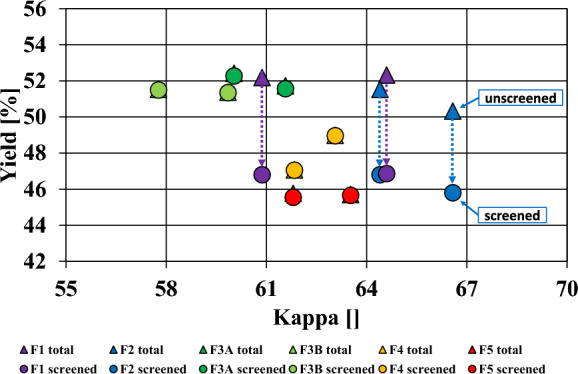
Screened/unscreened yield versus Kappa of the chip fractions: Triangles represent the total yield, dots represend screened yield; dashed arrows highlight the amount of yield that is lost through rejects. Two cooks per chip fraction were performed

Figure 7 shows the screened yield and total yield depicted over Kappa numbers. The results suggest that for the two cooks that were performed per chip fraction, the yield was significantly more stable compared to the Kappa. Also, the variation between the repeats is statistically not significant. Both of had to be expected from the repeatability trials. The total yield was almost identical to the screened yield for most fractions, except for the oversized (F1) and overthick chips (F2). Their total yield was equal to that of large accept (F3A) and small accept chips (F3B) (approximately 51–52% total yield). However, due to the high number of rejects, the screened yield of F1 and F2 was in the same order of magnitude as for the undersized fractions (rejects lowered the screened yield to approximately 46–47%). The pulp of F1 and F2 exhibited a higher Kappa than the accept fractions F3A and F3B. This is consistent with the literature that kraft pulping of large chips gives a high total yield, but also a high rejects content with a low screened yield and higher average Kappa (Colombo et al. 1964). The average yield loss due to rejects was 5.4% for the oversized fraction (F1) and 4.6% for the overthick fraction (F2).

The pins (F4) and fines (F5) produced barely any screenable rejects, so the total yield was equal to the screened yield. Both undersized fractions had a significantly lower screened yield compared to F3A or F3B. This was to be expected, as the small chip geometry should lead to overcooking of the material when cooked under the same cooking conditions (Morton et al. 2012; Quinde 2020a, b). Overcooking of smaller fractions dissolves more lignin from the wood, which should lead to a lower Kappa number, but also to a lower screened yield. However, the Kappa number of the pins and fines was higher than that of the small and large accept fractions. Also, the yield at equivalent Kappa should be equal or higher for the small chip fractions according to literature, which was not the case (Akhtaruzzaman and Virkola 1979a; Gullichsen et al. 1995).

In summary the higher Kappa and lower screened yield of the large fractions has been expected, as the inner parts of the chips are not sufficiently delignified. However, for the small fractions Kappa was higher than expected while exhibiting a lower yield at the same time. 

### Difficulties in kraft pulping of the fine fraction

**Fig. 8:**
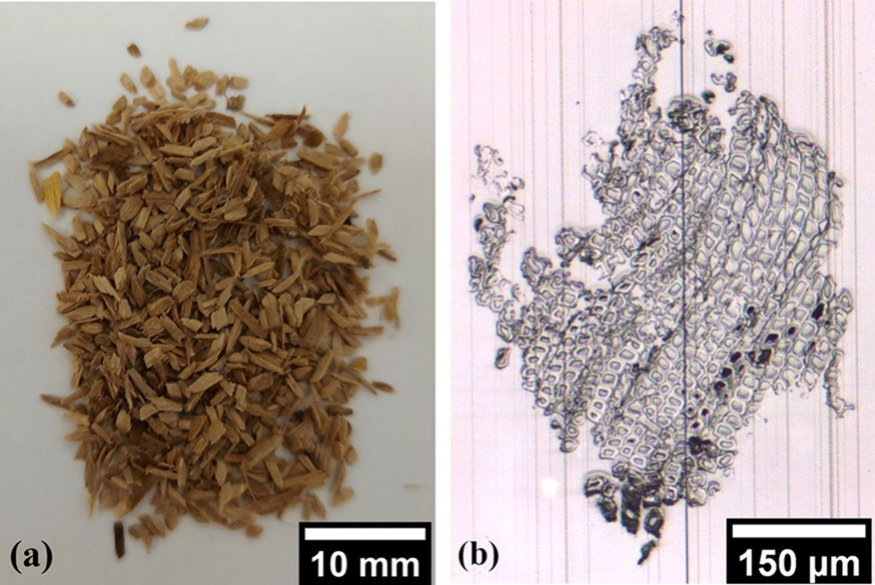
(**a**) Small, undercooked particles fractionated from fraction F5 using the Brecht-Holl fractionator with a 0.39 mm sieve; (**b**) microtome cutted particle image of the undercooked material

The kraft pulping of fines (F5) surprisingly turned out to be problematic. Small, undercooked particles (displayed in Fig. 8a) remained in the final pulp. It was not possible to separate these particles by using the 0.39 mm mesh sieve during screening. To further investigate these rejects and to exclude structural effects, microtome cutting of single particles from a batch like in Fig. 8a was performed. In Fig. 8b, an optical micrograph is depicted. Here, one can see that the fibers within such a reject particle are still closely bound together and show clear lumina. The thickness of the cell walls as well as the size of the lumina is varying, showing layers of earlywood and latewood, as expected. After kraft pulping of the fines (F5) in the rotary digester, these particles were found to be stuck on the surface and corners of the vessel. This undercooked material was most likely the result of insufficient mixing of cooking liquor and wood particles inside the rotary reactor. However, since the undercooked material in F5 should contain more lignin, it should have contributed to a higher total yield. This was not observed, leading to the investigation of the bark content in the chip fraction.

### Bark impurities in chip fractions

**Fig. 9:**
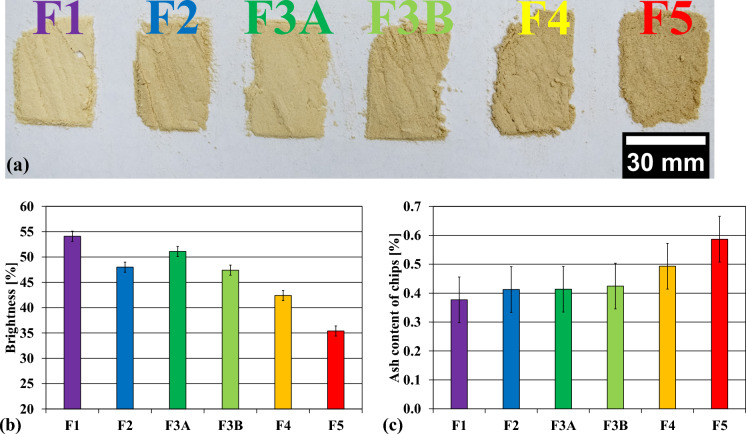
(**a**) powders obtained by grinding the chip fractions. From left to right: oversized-(F1), overthick- (F2), large accept- (F3A), small accept chips (F3B), pins (F4), fines (F5); (**b**) results of brightness measurements of the powders; (**c**) ash content of the chip fractions. The results represent one-time measurements of the well-mixed chip powder. The error bars represent the standard error of the measurement according to the standards (ISO 2470-2:2008 and Tappi T211)

After visual examination of the pins (F4) and fines (F5), a higher content of dark spots in the unprocessed material of those fractions became apparent. A higher bark content was thought to be the cause, but it was impossible to separate the dark particles from the smaller fractions without affecting the result. The bark of spruce contains a higher proportion of ash and lignin than stem wood (Timell and Ramalingam 1964). Samples of all chip fractions were collected and grounded in a Retsch rotor mill. A clear color difference was visible after the wood material was milled, as seen in Fig. 9a. The powder of larger chips exhibited the highest visual brightness while smaller fractions exhibited a darker tone. The only exception was the overthick fraction (F2), which was darker in appearance than the large accept chips (F3A). To quantify that, the powders were pressed into pellets and brightness measurements were done. The results of the analysis are presented in Fig. 9b and clearly indicate a brightness decrease with decreasing chip size, starting with a brightness of 54% of the oversized fraction (F1) and ending with 35% brightness of the fines fraction (F5). The overthick fraction (F2) deviates slightly from the brightness trend, which was also visible in the picture (Fig. 9a).

The results for the ash content are presented in Fig. 9c. The ash content of the fractions was increasing with decreasing chip size (0.38% ash in F1–0.59% ash in F5). Pins (F4) and fines (F5) had a significantly higher ash content compared to the other chips. The higher ash content and the color gradient of the chip powders are strong indicators for a higher bark content in smaller chip fractions. The brittle bark on larger chip fractions was probably crushed into smaller pieces due to the mechanical impacts during transport, pile storage, and chip handling. The small bark particles then accumulated in the smaller fractions during screening.

The kraft pulping of bark results in a higher Kappa number and a lower screened yield when cooked under the same conditions as wood chips (Miranda et al. 2012). Hence, the higher bark content in smaller chip fractions explains the higher Kappa at smaller yield in Fig. 7. Nevertheless, a different publication suggests that a greater concentration of bark should be found in thicker chip fractions. However, this study focused on hemlock wood rather than spruce (Becker 1992).

## Strength properties of chip fractions

**Fig. 10:**
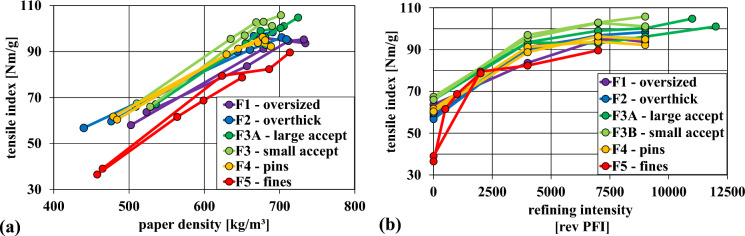
(**a**) tensile index versus density of the respective chip fractions at different beating degrees illustrated as dots (increasing refining intensity from left to right); (**b**) tensile strength as a function of refining intensity

According to the literature, the tensile strength should increase with decreasing chip thickness or increasing chip length (JHatton and Keays 1973; Akhtaruzzaman and Virkola 1980; Morton et al. 2012). Both, chip length and thickness decreased from F1–F5. Laboratory PFI refining of the pulps from the respective fractions was done and paper properties such as tensile strength measured. The tensile index as a function of paper density is presented in Fig. 10a. The individual data points are representing different degrees of PFI refining of the pulp, compare Fig. 10b. A higher tensile strength at a given density is favorable.

The undercooked particles of the fines pulp fraction had a considerable impact on the paper strength of the initial unrefined pulp. The solid particles, which were also visible in the produced paper, hindered fiber–fiber interactions and created weak spots in the paper. However, the difference between the pulp from fraction F5 and the other fractions became smaller with increasing refining intensity. In the process of refining the pulp, the undercooked particles in F5 were probably disintegrated, which allowed their fibers to properly contribute to the paper strength eventually. This was also apparent when looking at the paper produced after refining, which was much more homogeneous. Nonetheless, the tensile strength of fraction F5 was the weakest.

The obtained strength properties of fraction F4 were quite good, but still a bit lower than those of F3A and F3B. The small accept chips (F3B) emerged as the fraction with the highest paper strength, followed by the large accept fraction (F3A).

The tensile strength of the overthick fraction (F2) was very similar to that of the pins fraction (F4). The oversized fraction (F1) showed significantly lower paper strength than the other chip fractions, with the exception of the fine fraction.

Figure 10b depicts the tensile index at given refining intensity. Here it can be seen that the potential for strength development through refining is better for the small and large accept fractions (F3A & F3B). While the tensile index development of the oversized, overthick, pins and fines fractions has leveled out at approximately 7500 PFI revolutions, it appears that the strength properties of F3A and F3B can be further developed through refining.

**Fig. 11:**
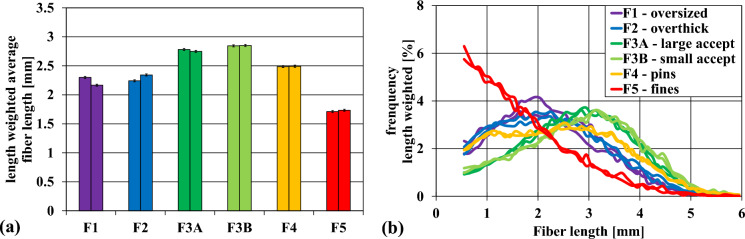
(**a**) Length-weighted average fiber length of the pulps from the respective chip fractions, (**b**) fiber length distribution

Overall, the strength properties of the pulp from fines (F5) were the worst. The pulp from the oversized fraction exhibited surprisingly low values, while the tensile index from overthick chips and pins was quite good. Both the large and small accept fractions yielded the highest paper strength. The cause for the observed paper strength of these fractions was probably their fiber length (see Fig. 11b).

### Fiber length distributions of industrial chip fractions

The breaking length or tensile index is related to the fiber length, among other effects (Wangaard and Woodson 1973). The fiber length of the pulp from all fractions was investigated. It was expected that the smaller the chip length, the more frequently the fibers were cut on average during the chipping process, and therefore the average fiber length should be shorter (Rydholm 1965; Akhtaruzzaman and Virkola 1979b). The results for the length-weighted average fiber length of the different pulps is presented in Fig. 11a. The course of the average fiber length reflected the results of the tensile strength measurement.

The large accept (F3A) and small accept fraction (F3B) exhibited the highest average fiber length with 2.78 and 2.84 mm respectively. The fines fraction (F5) had the shortest average fiber length with 1.73 mm, which was expected. Also, F4 (2.49 mm) was shorter than F3, which is plausible. Contrary to the expectations, the oversized fraction (F1) and the overthick fraction (F2) had a considerably smaller fiber length than F3—similar to of F4—despite these chips being larger than F3.

The fiber length distribution in Fig. 11b confirms a shift of fraction F1 and F2 to a lower fiber length. This means that the difference in average fiber length between the oversized- and accept fractions was not only due to a higher fines content. The frequency distribution of F4 revealed a greater proportion of large fibers in comparison to F1 and F2, although these two fractions consisted of much larger and thicker chips. The distribution of fiber length in the pins fraction follows a pattern more closely aligned with the acceptance fractions than F1 and F2, where a peak is observed at shorter fiber lengths. Hence, it is suggested that the variations in fiber length and tensile strength of F1 and F2 may be attributed to differences in wood composition rather than the chip size itself.

### Knots in industrial chip fractions

Akhtaruzzaman likewise observed in his dissertation that as chip thickness increased, fiber length and tensile strength decreased (Akhtaruzzaman and Virkola 1980). He pointed out that thicker chips from industrially chipped wood would experience more damage and fiber shortening during processing. But he also stated that industrially produced thick chips might contain higher amounts of heartwood, compression wood and branch wood, which influences the cooking behavior.

A visual examination of the wood raw material from the respective chip fractions gave the impression that the larger chips (F1 and F2) had a very high proportion of knots. The estimation of knots in smaller fractions proved difficult because visual differentiation from the small chips was impossible.

According to the publication of Axegård, more than 90% of the chips that contain knots are found in the oversized- and overthick chip fraction while less than 1% of accept fraction chips contain knots (Axegård 1978). In the work of Cáceres et al., the average chip thickness was correlated to the knot content in black spruce logs, they found that the average chip thickness increased with the knot content (Cáceres et al. 2016). In another paper the same group found that the density of white spruce knots was 2.4 times higher than the density of clear wood. Consequently, knots required up to eight times more cutting force than clear wood (Cáceres et al. 2018). This is likely to be a key factor in why the oversized- and overthick chips are created during the chipping process in the first place.

The described high density of the knots is affecting the impregnation- and cooking behavior (Ulla Sahlberg 1995), which additionally contributes to the high reject content for the oversize/overthick fractions F1 and F2 (see dashed lines in Fig. 7). The produced rejects themselves seemed to have a knot-like appearance.

Brändström's measurements of the fiber properties of Norway Spruce showed that the fiber length of the knots (1.3 mm) was 50% shorter than that of the stem wood (Brändström et al.). Even the so-called knotty wood (wood surrounding the knots) exhibited a shorter fiber length (2.0 mm) than the stem wood (2.6 mm). In conclusion the lower fiber length in knots and knotty wood explains the lower fiber length of the oversized (F1) and overthick chips (F2) fractions, and thus also their lower tensile index (see Figs. 10 and 11).

Although a high number of knots in the oversized- and overthick fraction has been discussed, the direct quantitative analysis of knot content in different chip fractions was found to be challenging. However, future analysis might be facilitated by chemical analysis, exploiting the considerably high lignan content in knots (Holmbom et al. 2003).

### Process alternatives and potential for undersized- or oversized chips

The analysis of the chip fractions has shown that there is a rather high proportion of pins (F4), fines (F5), overthick- (F2) and oversized (F1) chips in the industrial process even after screening (see Fig. 5). When these fractions are cooked together with the accept fraction under the same conditions, some potential is certainly lost.

### Undersized chips

Processing smaller chip sizes results in yield losses due to overcooking (Quinde 2020a, b), which could be counteracted by cooking them in a separate digester to achieve similar kappa and yield (Gullichsen et al. 1995). While F5 produces clearly lower quality pulp, the strength potential from the pin chips F4 is nearly as good as the pulp of the accept fractions (see Fig. 10). It would be possible to cook only F5, but also F4 in a separate digester. Sawdust and pins from sawmill may be added as additional cheap raw material here. However, the accumulation of bark in smaller chip fractions could increase dark spots in the pulp and paper strength will suffer due to the shorter fiber length (see Fig. 11). The separation of pins and fines from the chips entering the main digester could potentially increase the process stability and production output by decreasing digester disturbances such as channeling, hang ups or screen clogging (Quinde 2020a, b; Rahman et al. 2022). The resulting pulp should also have higher quality.

### Oversized chips

Usually, oversized chips are rechipped or crushed. During rechipping a substantial number of pins and fines are formed (Kreft and Javid 1989). The crushing of chips led to a lower reject content while keeping the pins and fines generation at a low level (MacLeod and Dort 2005). They also observed a lower paper strength and fiber length, which they attributed to mechanical fiber damage. Following Brändström’s findings (Brändström et al. 2005) and our results (see Figs. 10 and 11), we attribute the observed lower fiber length and paper strength to the high knot content in the overthick and oversized fraction. Chips from F1 and F2 are hence reducing the quality of the pulp even after rechipping or crushing. The high wood density (Cáceres et al. 2018) and the elevated lignin content of knots furthermore reduces pulp quality due to higher shive content. Thus, it could be an option to also cook shredded oversized chis in a separate digester, reducing rejects and chemical consumption, and increasing yield.

The yield of F1 and F2 could be increased by 5.4 and 4.6%, respectively, provided no rejects are generated when cooking the shredded knots.

Finally, it would be possible to join F5, F4 and the rechipped F1 and F2 to produce a lower quality pulp fraction for applications where the mechanical performance of the pulp is less relevant. At the same time the main fraction can be produced at higher efficiency and quality in the main digester.

The benefits of such a process needs to be proven, e.g., by additional experiments combined with a LCA, but has to be left for future work to come.

## Conclusions

### Low screening efficiency in the industrial environment

In the pulp mill investigated, there are large proportions of oversized and undersized chips due to the poor separation efficiency of the post-chipping screening. These non-ideally sized chips enter the digester together with the optimized chips and cause deviations in the process quality. The mill is a modern mill in Europe with state-of-the-art equipment. It can thus be suspected that poor chip screening efficiency is also common for other pulping operations worldwide. Increasing the screening efficiency and separating the fines fraction F5 (for incineration) and the oversize fractions F1 and F2 for rechipping should bear potential for a low CAPEX investment, has the potential for cost savings (yield increase, reject reduction) and quality increase.

### Pulp quality of over- and undersized chips

Cooking smaller chip fractions, i.e., pins (F4) and fines (F5), we expectedly (Gullichsen et al. 1992) found lower screened yields but unexpectedly (Akhtaruzzaman and Virkola 1979a) found higher kappa values compared to the accept fractions (see Fig. 7). Due to high ash content and lower brightness in F4 and F5 we attributed this to a higher bark content. The brittle bark on the larger chips probably broke into smaller pieces due to mechanical interactions and accumulated in smaller fractions. While the fraction F5 gives really low quality pulp, the properties of F4 are only moderately deteriorated compared to the accept fraction.

Thicker chip fractions, i.e., oversize (F1) and overthick (F2) chips, had considerably lower tensile strength than the accept fractions, as reported in the literature (Akhtaruzzaman and Virkola 1980). Being not fully cooked due to their oversize, they produced a significant amount of screening reject. Unexpectedly, the average fiber length was substantially shorter. A visual inspection of chips and literature (Axegård 1978) suggest a much higher content of knots. The inherent characteristics of knots, such as short fiber length, higher lignin content, and high density, are explaining the lower pulp quality. So knots are, first, leading to oversized chips in chipping and, second, even at correct size to insufficient defiberation in pulping and lower fiber length.

### Process potential

It can be considered to cook the undersized fractions F4 and F5 separately, obtaining a lower quality pulp fraction but process- and quality improvements for the main fraction and an overall yield increase. Also, the oversized fractions F1 and F2 could be cooked separately after rechipping, also obtaining a lower quality pulp fraction and process- and yield improvements in the main process. It might be considered to combine undersized and rechipped oversized fractions to a lower quality pulp fraction. This is straightforward to implement for batch cooking mills, which are not so common. For continuous digesters this would require investing into a separate, smaller digester. That is a major investment that probably is only economically feasible if de-bottlenecking of the continuous digester is required in the mill.

The key requirement for all the possible measures suggested above, of course would be a higher separation efficiency in the poorly working chip screening process. Suggesting that our findings can be transferred to other mills there might be a lot of potential for improvements in yield, process runnability and pulp quality by implementing and monitoring a high separation efficiency in chip screening.

## Data Availability

All data that was analyzed for this study is available from the corresponding author on reasonable request.

## Code availability

Commercial software was used.

## Declarations

### Human and animal participants

No studies on human or animal participants were performed.
